# Dataset on gene expression in the elderly after Mindfulness Awareness Practice or Health Education Program

**DOI:** 10.1016/j.dib.2018.03.086

**Published:** 2018-03-26

**Authors:** Hwee-Woon Lim, Woei-Yuh Saw, Lei Feng, Yuan-Kun Lee, Ratha Mahendran, Irwin Kee-Mun Cheah, Iris Rawtaer, Alan Prem Kumar, Ee-Heok Kua, Rathi Mahendran, Ene-Choo Tan

**Affiliations:** a KK Research Laboratory, KK Women's and Children's Hospital, Singapore; b Saw Swee Hock School of Public Health, National University of Singapore, Singapore; c Life Sciences Institute, National University of Singapore, Singapore; d Department of Psychological Medicine, Yong Loo Lin School of Medicine, National University of Singapore, Singapore; e Department of Microbiology and Immunology, Yong Loo Lin School of Medicine, National University Hospital, Singapore; f Department of Surgery, Yong Loo Lin School of Medicine, National University of Singapore, Singapore; g Department of Biochemistry, Yong Loo Lin School of Medicine, National University of Singapore, Singapore; h Medical Science Cluster, Yong Loo Lin School of Medicine, National University of Singapore, Singapore; i Cancer Science Institute of Singapore, National University of Singapore, Yong Loo Lin School of Medicine, National University of Singapore, Singapore; j Duke-NUS Medical School, Singapore; k SingHealth Duke-NUS Paediatrics Academic Clinical Program, Singapore

**Keywords:** Elderly, Gene expression, Mild cognitive impairment, Mindfulness Awareness Practice, Health education program

## Abstract

It has been reported that relaxation techniques can improve physical health and cognitive function. A number of studies involving different types of relaxation practices showed changes in expression of genes. We investigated the gene expression pattern of a cohort of elderly subjects of Asian descent after weekly (for the first three months) and monthly (for the subsequent six months) intervention. Sixty consenting elderly subjects (aged 60–90 years) with mild cognitive impairment were assigned to either the Mindfulness Awareness Practice (MAP) or Health Education Program (HEP) group in a randomized controlled trial to assess the effectiveness of the programs in preventing further cognitive decline and evaluate the influence on neurological, cellular and biochemical factors. Blood samples were collected before the start of intervention and after nine months for gene expression profiling using Affymetrix Human Genome U133 Plus 2.0 arrays. The dataset is publicly available for further analyses.

## Specifications Table

**Table:** 

**Subject area**	Biology
**More specific subject area**	Gene expression
**Type of data**	Tables and figures
**How data was acquired**	Affymetrix GeneChip Human Genome U133 Plus 2.0 Arrays, Microarray Suite 5, Robust Multi-Array Average and statistical analysis
**Data format**	Raw (CEL.) and normalized (CHP.)
**Experimental factors**	Pre-intervention samples collected before the start of each program, post-intervention samples collected after 9 months of the program
**Experimental features**	Subjects were randomized to one of two groups. One group was taught Mindfulness Awareness Practice techniques (MAP). The second group underwent the Health Education Program (HEP) under which they received talks on healthy living topics.
**Data source location**	National University of Singapore and Research Laboratory, KK Women's and Children's Hospital, Singapore
**Data accessibility**	http://www.ncbi.nlm.nih.gov/geo/query/acc.cgi?acc=GSE108215

## Value of the data

• Practices like yoga, Tai Chi, breathing exercises, and meditation are thought to evoke the relaxation response that counteracts stress response [1].
• A small study of experienced practitioners showed that mindfulness meditation could influence expression of genes related to histone modifications and pro-inflammatory genes [2]. There is also report that after listening to relaxation response-eliciting or health education recordings, healthy adults showed enhanced expression of genes associated with metabolism and telomere maintenance, while the expression of genes linked to inflammatory response and stress-related pathways was reduced [3].
• We investigated whether Asian elderly subjects with mild cognitive impairment would show gene expression changes after undergoing nine months of mindfulness practice program or health education program.
• The identification of genes and pathways can be used for the development of markers to assess the effectiveness of the mindfulness practice or other relaxation exercises.

## 1 Data

### 1.1 Isolation of RNA from blood and globin depletion

The average amount of RNA extracted was 14.88 µg while one sample collected before initiation of HEP intervention had insufficient RNA for further analysis. After going through GLOBINclear, the yield was between 0.46 and 1.79 µg, with RNA integrity numbers of between 6.6 and 8.9, and 260/280 absorbance ratios of between 1.8 and 2.0 µg.

### 1.2 Microarray gene expression analysis

**Fig. 1:**
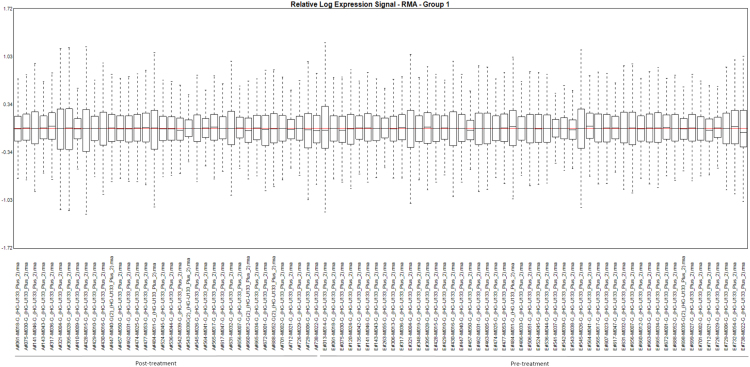
Relative Log Expression plot based on RMA-normalized signal data. Each boxplot represents a sample. The boxplots presenting the distribution of the log-ratios are centered near 0 and have similar spread.

**Fig. 2:**
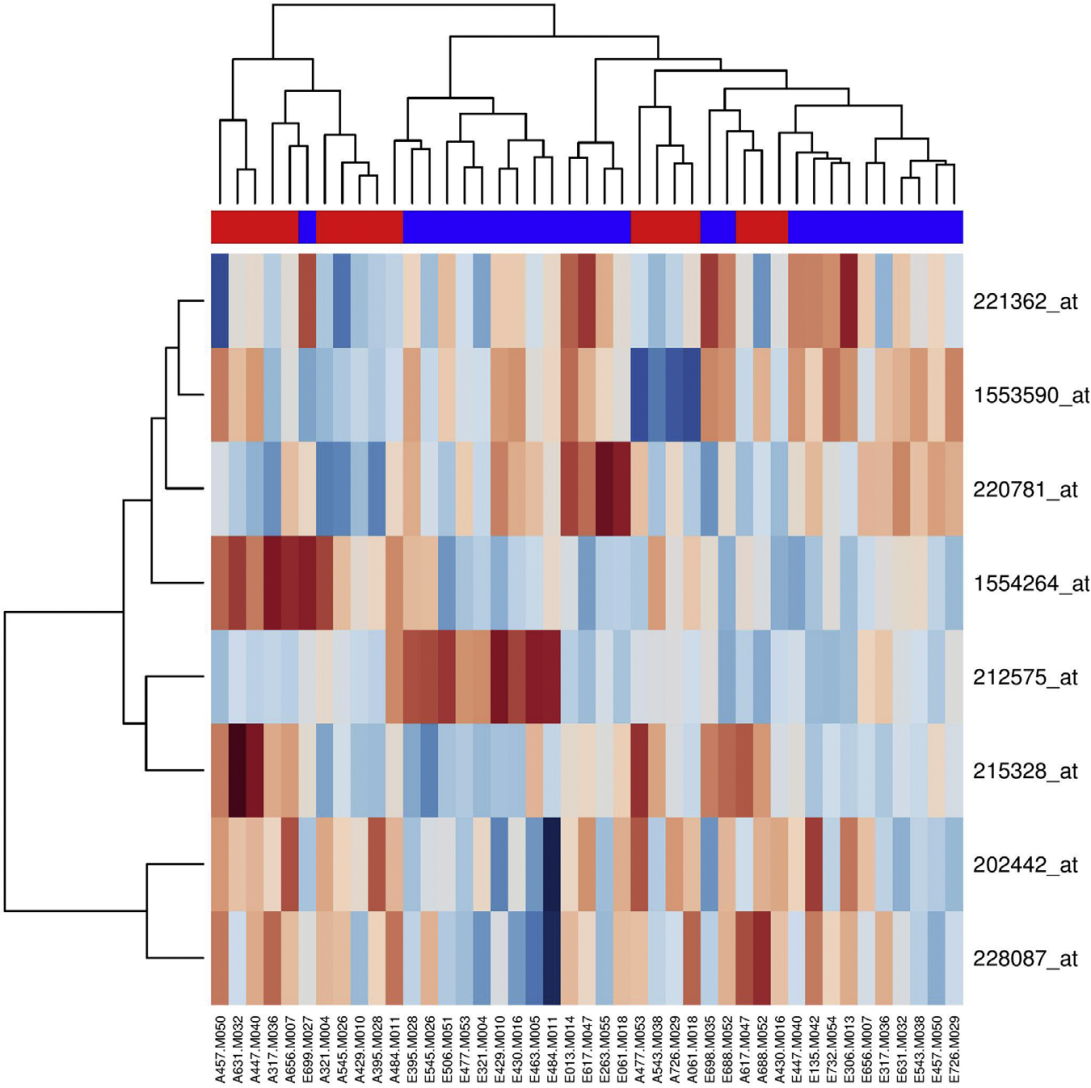
Heatmap and dendrogram of the differential gene expression profiles for the eight gene markers generated from unmatched pre-MAP and post-MAP intervention samples using MAS5.0 normalization before FDR correction. The pre-intervention group is colored in blue and post-intervention group is in red. The relative expression level of each gene among all the tested samples have been assigned sequentially from dark red color to dark blue color in accordance with low to high expression level.

**Fig. 3:**
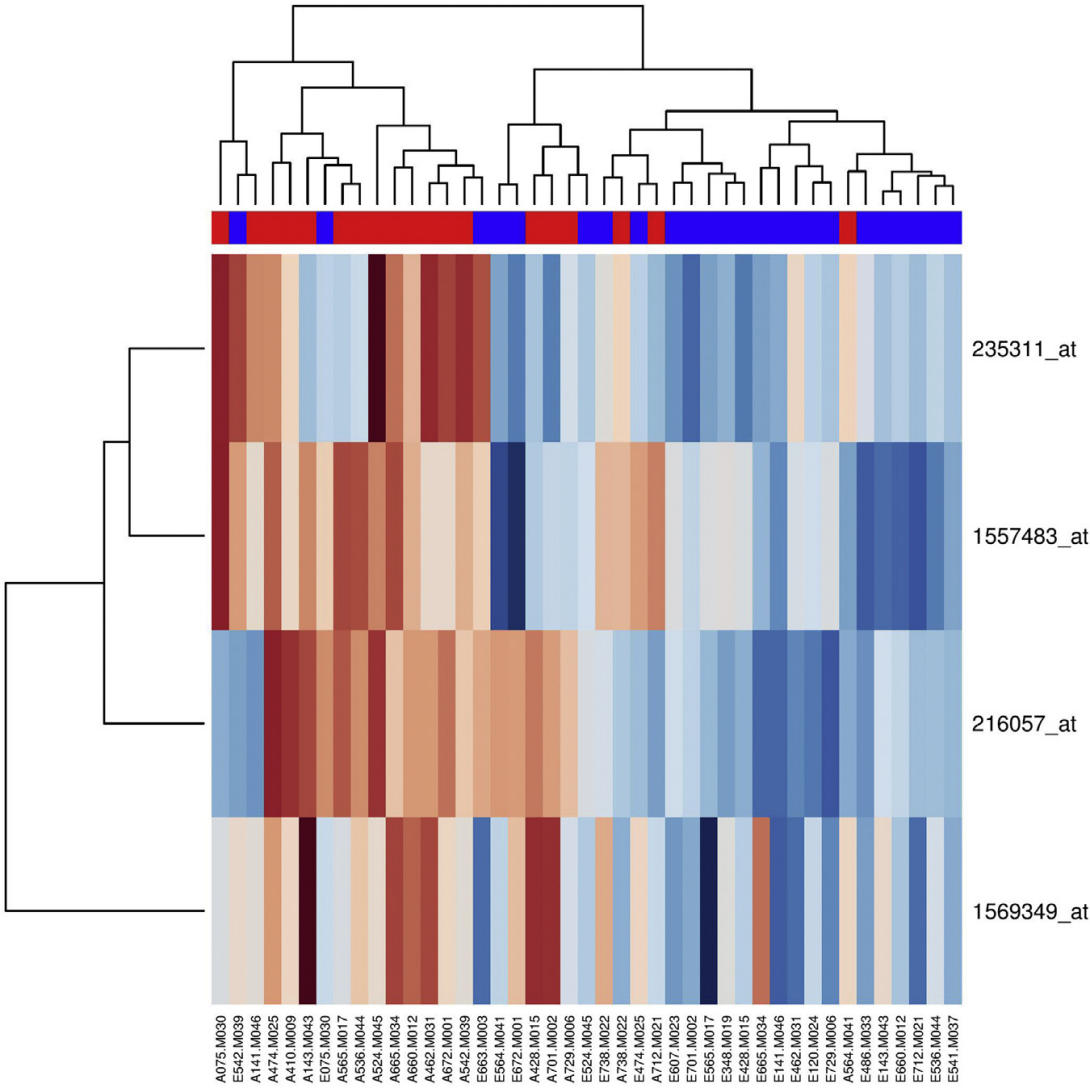
Heatmap and dendrogram of the differential gene expression profiles for the four gene markers generated from unmatched pre-HEP and post-HEP intervention samples using MAS5.0 normalization before FDR correction. The pre-intervention group is colored in blue and post-intervention group is in red. The relative expression level of each gene among all the tested samples have been assigned sequentially from dark red color to dark blue color in accordance with low to high expression level.

Relative Log Expression (RLE) of all the samples was at equal level, signal intensity across the samples were also comparable (Fig. 1). For the pre-MAP and post-MAP analysis using Microarray Suite 5 (MAS5) normalization with log2 transformed, the eight probesets with *p*-value < 0.0005 (before FDR correction) from unmatched samples analysis are listed in Table 1, and the gene expression heatmap in Fig. 2. The corresponding result for the matched samples analysis between pre-MAP and post-MAP is shown in Table 2. For the group with HEP intervention, the list of probesets which had *p*-value < 0.0005 (before FDR correction) from unmatched samples analysis is presented in Table 1, with the heatmap of the gene expression profiles shown in Fig. 3. The corresponding matched samples analysis result between pre-HEP and post-HEP samples is presented in Table 2.

**Table 1:** Genes with differentially expressed probesets from unmatched samples analysis using MAS5 normalization.

**Group**	**Gene symbol**	**Probesets**	*p**before FDR**	*p**after FDR**	a**Fold change**
**MAP**	*DEC1*	220781_at	5.83E − 05	0.75	3.34
Pre: *n* = 26	*CCDC126*	228087_at	6.74E − 05	0.75	0.83
Post: *n* = 17	*HTR5A*	221362_at	9.18E − 05	0.75	2.08
	*TMEM259*	212575_at	1.01E − 04	0.75	2.69
	*CKAP2*	1554264_at	1.42E − 04	0.84	0.32
	*FAM27E2/E3*	1553590_at	1.84E − 04	0.91	2.22
	*EFR3B*	215328_at	3.44E − 04	0.99	0.39
	*AP3S1*	202442_at	4.44E − 04	0.99	0.89
**HEP**	*RAB3GAP2*	216057_at	1.53E − 04	0.99	0.28
Pre: *n* = 24	LOC284788	1557483_at	1.54E − 04	0.99	0.41
Post: *n* = 19	*C11orf30*	1569349_at	1.55E − 04	0.99	0.71
	*FKBP14*	235311_at	2.92E − 04	0.99	0.39

FDR = false discovery rate.

* *p* from ANCOVA after adjusting for batch and gender effects.

a Batch and gender-adjusted fold change.

**Table 2:** Genes with differentially expressed probesets from matched samples analysis using MAS5 normalization.

**Group**	**Gene symbol**	**Probesets**	*p**before FDR**	*p**after FDR**	a**Fold change**
**MAP**	*DEC1*	220781_at	4.70E − 06	0.14	3.77
Pre: *n* = 17	*TEF*	215673_at	8.61E − 05	0.99	0.24
Post: *n* = 17	*CKAP2*	1554264_at	2.79E − 04	0.99	0.30
	*CCDC126*	228087_at	3.69E − 04	0.99	0.82
	*TTLL7*	219882_at	3.92E − 04	0.99	2.85
	*LRRC37A4P*	229821_at	4.77E − 04	0.99	1.28
**HEP**	*GRIK2*	215655_at	1.73E − 04	0.99	0.19
Pre: *n* = 18	*PRKCZ*	1569748_at	1.82E − 04	0.99	3.16
Post: *n* = 18	*SLC2A5*	230705_at	2.42E − 04	0.99	0.37
	LOC284788	1557483_at	2.98E − 04	0.99	0.38
	*SCFD1*	233229_at	3.04E − 04	0.99	0.60

FDR = false discovery rate.

* *p* from ANCOVA after adjusting for batch effect.

a Batch-adjusted fold change.

The list of probesets with *p*-value < 0.0005 (before FDR correction) between pre- and post-intervention samples within each intervention group from the analysis using Robust Multi-array Average (RMA) normalization are presented in Table 3 for unmatched samples analysis whereas Table 4 shows the corresponding matched samples analysis.

**Table 3:** Genes with differentially expressed probesets from unmatched samples analysis using RMA normalization.

**Group**	**Gene symbol**	**Probesets**	*p**before FDR**	*p**after FDR**	a**Fold change**
**MAP**	LOC101928211	1559930_at	7.09E − 06	0.21	1.14
Pre: *n* = 26	*B3GNT2*	224154_at	2.31E − 04	0.99	1.17
Post: *n* = 17	LOC100996251	1568892_at	4.44E − 04	0.99	0.88
	*CSNK1A1*	205764_at	4.89E − 04	0.99	0.85
**HEP**	*SNAP91*	204953_at	1.00E − 04	0.99	1.14
Pre: *n* = 24	*SRSF4*	241245_at	1.28E − 04	0.99	0.86
Post: *n* = 19	*FABP6*	210445_at	4.00E − 04	0.99	1.20
	*KCNB2*	208123_at	4.32E − 04	0.99	1.15

FDR = false discovery rate.

* *p* from ANCOVA after adjusting for batch and gender effects.

a Batch and gender-adjusted fold change.

**Table 4:** Genes with differentially expressed probesets from matched samples analysis using RMA normalization.

**Group**	**Gene symbol**	**Probesets**	*p**before FDR**	*p**after FDR**	a**Fold change**
**MAP**	LOC101928211	1559930_at	5.01E − 05	0.99	1.16
Pre: *n* = 17	*PLA2G16*	235110_at	1.49E − 04	0.99	1.24
Post: *n* = 17					
**HEP**	*RPL23AP53*	222225_at	9.42E − 05	0.99	0.81
Pre: *n* = 18					
Post: *n* = 18					

FDR = false discovery rate.

* *p* from ANCOVA after adjusting for batch effect.

a Batch adjusted fold change.

**Fig. 4:**
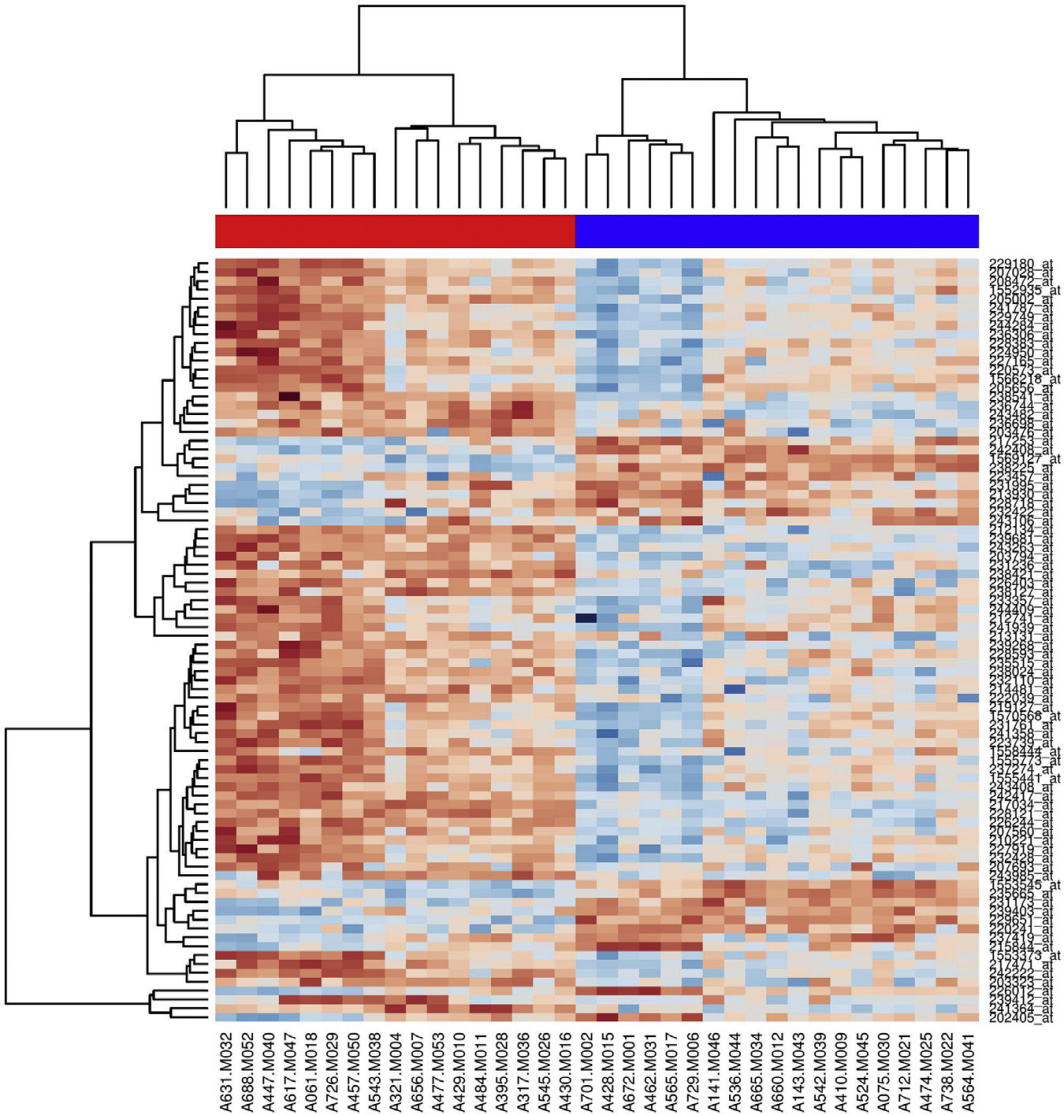
Heatmap and dendrogram of the differential gene expression profiles for 86 gene markers with at least 1.5 fold up- or down- regulation, generated from post-intervention HEP and MAP samples using RMA normalization. The HEP group is colored in blue and MAP group is in red. The relative expression level of each gene among all the tested samples have been assigned sequentially from dark red color to dark blue color in accordance with low to high expression level.

Additional comparison was made between post-MAP and post-HEP samples, the 86 significant probesets with *p*-value < 0.05 (after FDR correction) or fold change cut-off (fc > 1.5 or fc < 0.66) are listed in Table 5. The heatmap for the corresponding gene expression profiles is shown in Fig. 4.

**Table 5:** Genes with differentially expressed probesets between post-MAP (*n* = 17) and post-HEP (*n* = 19) from unmatched samples analysis using RMA normalization.

**Gene symbol**	**Probesets**	*p**before FDR**	*p**after FDR**	a**Fold change**
*LOC101928457*	217034_at	1.51E − 15	4.49E − 11	0.57
*AHDC1*	205002_at	7.54E − 14	1.12E − 09	0.51
*ENTHD2*	239681_at	1.40E − 13	1.39E − 09	0.62
*MIR6716///PHLDB1*	212134_at	1.46E − 12	1.08E − 08	0.63
*CLEC14A*	226244_at	8.10E − 12	4.80E − 08	0.60
*LINC00482*	243263_at	6.88E − 11	2.27E − 07	0.66
*NDUFS1*	239268_at	1.12E − 10	3.02E − 07	0.65
*C9orf172*	236744_at	3.01E − 10	6.86E − 07	0.59
*GALNT5*	232110_at	3.30E − 10	7.00E − 07	0.58
*LOC101928104*	217471_at	3.76E − 10	7.44E − 07	0.61
*TGFB2*	228121_at	4.81E − 10	8.40E − 07	0.55
*WWC1*	229180_at	1.16E − 09	1.73E − 06	0.60
*IKZF4*	208472_at	1.74E − 09	2.15E − 06	0.64
*KLK14*	220573_at	2.64E − 09	2.80E − 06	0.56
*LOC283278///PLEKHA7*	242417_at	3.69E − 09	3.25E − 06	0.63
*UBA6*	1555441_at	4.88E − 09	3.92E − 06	0.64
*CDC42BPA*	203794_at	6.07E − 09	4.40E − 06	0.62
*MYCNOS*	207028_at	6.46E − 09	4.46E − 06	0.61
*C21orf58*	238541_at	6.37E − 09	4.46E − 06	0.63
*GAS6-AS1*	238127_at	6.81E − 09	4.52E − 06	0.51
*BPIFC*	1555773_at	7.86E − 09	4.95E − 06	0.65
*TMC4*	226403_at	8.09E − 09	4.95E − 06	0.61
*LINC01106///LINC01123*	242222_at	1.37E − 08	6.99E − 06	0.61
*WDR64*	1553373_at	1.98E − 08	9.77E − 06	0.65
*ZCCHC5*	1552935_at	2.37E − 08	1.00E − 05	0.62
*SLC28A1*	207560_at	2.37E − 08	1.00E − 05	0.64
*SYNE4*	235515_at	2.31E − 08	1.00E − 05	0.63
*AC100830.4*	238024_at	6.99E − 08	2.38E − 05	0.65
*MTMR9LP*	228593_at	8.74E − 08	2.76E − 05	0.66
*KIAA2026*	236306_at	9.68E − 08	2.90E − 05	0.64
*LINC01208*	237274_at	9.62E − 08	2.90E − 05	0.65
*LOC101927703*	241787_at	9.94E − 08	2.92E − 05	0.63
*LOC100130502*	1570568_at	2.27E − 07	5.26E − 05	0.61
*CHRNA3*	210221_at	2.45E − 07	5.47E − 05	0.64
*UCA1*	227919_at	3.51E − 07	6.92E − 05	0.64
*PNPLA7*	228383_at	3.67E − 07	7.02E − 05	0.65
*RC3H2*	238421_at	4.32E − 07	8.12E − 05	0.66
*ANO4*	229749_at	5.52E − 07	9.63E − 05	0.63
*RP11-805I24.3*	244284_at	6.59E − 07	1.12E − 04	0.62
*PADI1*	223739_at	7.83E − 07	1.28E − 04	0.61
*MOGAT2*	232428_at	8.68E − 07	1.37E − 04	0.64
*KIF18B*	222039_at	1.10E − 06	1.62E − 04	0.63
*RP11-774O3.1///RP11-774O3.2*	243408_at	1.12E − 06	1.63E − 04	0.65
*PTGFRN*	224950_at	1.29E − 06	1.83E − 04	0.61
*FFAR1*	231761_at	1.30E − 06	1.84E − 04	0.58
*TMEM57*	241364_at	1.86E − 06	2.39E − 04	0.54
*HIST1H2AM*	214481_at	2.39E − 06	2.76E − 04	0.63
*EPS15L1*	243482_at	2.48E − 06	2.82E − 04	0.61
*SKA3*	227165_at	3.09E − 06	3.22E − 04	0.65
*WFIKKN2*	241358_at	4.44E − 06	4.13E − 04	0.65
*PRR15L*	219127_at	5.80E − 06	4.99E − 04	0.61
*ZFP57*	231236_at	1.17E − 05	8.33E − 04	0.61
*CCDC154*	244409_at	1.35E − 05	9.29E − 04	0.66
*PCDH17*	205656_at	2.55E − 05	1.45E − 03	0.65
*CAV2*	203323_at	2.74E − 05	1.52E − 03	0.65
*GTF2A2*	243985_at	4.30E − 05	2.10E − 03	0.65
*KRTAP5-2*	1566218_at	1.04E − 04	3.85E − 03	0.66
*DYNC1I2*	236698_at	1.05E − 04	3.87E − 03	0.63
*IRF5*	239412_at	1.41E − 04	4.70E − 03	0.46
*TPBG*	203476_at	1.76E − 04	5.41E − 03	0.58
*CACNB4*	207693_at	1.90E − 04	5.75E − 03	0.65
*TRIM67*	233357_at	2.05E − 04	6.03E − 03	0.60
*IQGAP3*	241939_at	2.72E − 04	7.43E − 03	0.62
*DQ592442*	1558444_at	2.78E − 04	7.53E − 03	0.58
*MAOA*	212741_at	1.17E − 03	1.97E − 02	0.60
*OLFM1*	213131_at	4.26E − 03	4.62E − 02	0.56
*MIR146A*	238225_at	4.60E − 11	1.71E − 07	1.67
*PYROXD1*	231173_at	1.49E − 09	2.04E − 06	1.91
*SEZ6*	229651_at	2.07E − 09	2.33E − 06	1.53
*ILDR1*	1553545_at	2.90E − 09	2.87E − 06	1.59
*ATG12*	213930_at	3.00E − 09	2.87E − 06	1.76
*TMCO3*	220241_at	3.84E − 09	3.25E − 06	1.66
*ANKRD11*	226012_at	5.33E − 09	4.09E − 06	1.60
*RP11-876N24.5*	1569127_at	1.00E − 08	5.82E − 06	1.66
*CCDC120*	239403_at	1.29E − 08	6.83E − 06	1.55
*STYX*	242408_at	2.63E − 08	1.09E − 05	1.57
*SH3BP2*	217253_at	7.30E − 08	2.46E − 05	1.50
*TIAL1*	202405_at	2.68E − 07	5.70E − 05	1.69
*AC018766.6*	235665_at	1.07E − 06	1.61E − 04	1.57
*TNPO2*	215844_at	3.07E − 05	1.66E − 03	1.51
*GGACT*	232422_at	4.35E − 05	2.11E − 03	1.67
*CAAP1*	231995_at	3.16E − 04	8.15E − 03	1.55
*RP11-722E23.2*	237419_at	9.47E − 04	1.70E − 02	1.55
*CLEC12A*	243106_at	1.14E − 03	1.94E − 02	2.06
*COPG2*	223457_at	3.34E − 03	3.96E − 02	1.51
*ZNF44*	228718_at	3.58E − 03	4.12E − 02	1.50

FDR = false discovery rate.

* *p* from ANCOVA after adjusting for batch and gender effects.

a Batch and gender-adjusted fold change.

### 1.3 Pathway analysis

Analysis using *The Database for Annotation, Visualization and Integrated Discovery* (DAVID) version 6.8 showed that *RAB3GAP2, GRIK2, SCFD1* which are altered following HEP intervention are associated with disorders related to HDL cholesterol according to the data from the Genetic Association Database (GAD), (Table 6). *GRIK2* is also found to be involved in other GAD diseases including echocardiography, triglycerides, tobacco use disorder, autism, schizophrenia, and other psychiatric disorders. None of the probesets is related to any disease in the Online Mendelian Inheritance in Man (OMIM) database. Kyoto Encyclopedia of Genes and Genomes (KEGG) pathway analysis indicates that *GRIK2* and *SLC2A5* which were upregulated for the HEP group are involved in neuroactive ligand-receptor binding (Table 7).

**Table 6:** Genes involved in GAD disease.

**GAD disease**	**Probesets**	**Gene**	*p**-value**
Cholesterol, HDL	216057_at	*RAB3GAP2*	0.042
	215655_at	*GRIK2*	
	233229_at	*SCFD1*	
			
Tobacco Use Disorder	1569349_at	*C11orf30*	0.188
	215655_at	*GRIK2*	
	202442_at	*AP3S1*	
	233229_at	*SCFD1*	
	230705_at	*SLC2A5*	
			
Several psychiatric disorders	215655_at	*GRIK2*	0.208
	221362_at	*HTR5A*	
			
Autism	215655_at	*GRIK2*	0.244
	221362_at	*HTR5A*	
			
Echocardiography	215655_at	*GRIK2*	0.261
	233229_at	*SCFD1*	
			
Triglycerides	215655_at	*GRIK2*	0.290
	233229_at	*SCFD1*	
			
Schizophrenia	215655_at	*GRIK2*	0.464
	221362_at	*HTR5A*	

Thresholds set at minimum count of 2 and maximum EASE score (*p*-value) of 0.5.

* *p*-value of a modified Fisher's exact test (EASE score) from DAVID.

**Table 7:** Genes involved in KEGG pathway.

**KEGG pathway**	**Probesets**	**Gene**	*p**-value**
Neuroactive ligand-receptor interaction	215655_at,	*GRIK2*	0.12
	221362_at	*HTR5A*	

Thresholds set at minimum count of 2 and maximum EASE score (*p*-value) of 0.5.

* *p*-value of a modified Fisher's exact test (EASE score) from DAVID.

## 2 Experimental design, materials and methods

### 2.1 Study design

The study protocol was approved by the National University of Singapore Institutional Review Board. Sixty elderly who met the criteria below were enrolled:

• Between 60 and 85 years old.
• Living in the community.
• Fulfilled the operational criteria/definition of mild cognitive impairment including at least one age-education adjusted neuropsychological test Z score < − 1.5, did not meet DSM-IV criteria for dementia, had memory or cognitive complaint which was corroborated by a reliable informant and had intact activities of daily living.
• Could function independently.
• Able to travel on their own to participate in the intervention programme.
• Did not have a neurological condition such as epilepsy or Parkinson's disease.
• Did not have a major psychiatric condition such as major depressive disorder.
• Did not suffer from a terminal illness at the time of enrollment.
• Had no marked upper and lower limb motor difficulties.
• Not participating in another interventional study at the same time.

### 2.2 Intervention

Participants in the MAP group were taught mindfulness awareness practice techniques modelled on McBee [4]. All participants gathered in a group once a week for 40 min for the first 12 weeks, followed by once a month for 45 min for the subsequent 6 months. They were required to practise these techniques at home daily and keep a record of their practice.

The HEP participants attended weekly talks on healthy living topics such as hypertension, diabetes, dementia, depression, medications, exercise, diet, sleep, home safety, falls and social support. The sessions were led by an instructor who had experience conducting similar teaching sessions for the elderly. Participants were provided with hand-outs at each session and were also required to keep a record of activities to complete in-between sessions. The sessions were weekly for 40 min for the first 12 weeks, and then once a month for 6 months.

### 2.3 Samples and RNA preparation

Blood samples (1–3 ml) were collected in Tempus Blood RNA Tube from 51 subjects before the start of intervention (pre-intervention samples) and 36 subjects after nine months (post-intervention samples). The blood tubes were stored in the freezer at − 80 °C and processed in batches. Total RNA was extracted using Tempus Spin RNA Isolation Kit (Applied Biosystems, CA, USA). The purity and concentration of the total RNA were determined by UV–vis Spectrophotometer (Quawell, CA, USA) and Quantus Fluorometer (Promega, WI, USA). Globin mRNA depletion was performed with a minimum of 800 ng of total RNA using the GLOBINclear Kit (Ambion, TX, USA). The purity and concentration of the GLOBINclear RNA were determined by UV–vis Spectrophotometer and Quantus Fluorometer, respectively. The integrity of the GLOBINclear RNA was evaluated by RNA ScreenTape assay (Agilent Technologies, Waldbronn, Germany).

### 2.4 Microarray and quality control

Eighty-seven samples (51 pre-intervention, 36 post-intervention) were processed in batches of 4–12 samples. cRNA was synthesised from 100 ng of GLOBINclear RNA using GeneChip 3’ IVT PLUS Reagent Kit according to the manufacturer's protocol. Fragmented and labeled cRNA (11 µg) was hybridised to Affymetrix GeneChip Human Genome U133 Plus 2.0 arrays according to the manufacturer's protocol. Arrays were scanned using the Affymetrix GeneChip 3000 7G with autoloader and the captured images were analyzed using Affymetrix GeneChip Command Console version 4.3.2. Metric analysis was carried out according to the instructions provided by manufacturer using the Affymetrix Expression Console 1.4.1.46. Relative Log Expression (RLE) generated from RMA normalization using Affymetrix Expression Console was used to ensure the compatibility across the all samples and identify the outliers.

All available data were used to compare the difference in gene expression between pre- and post-intervention for unmatched samples analysis of each intervention group. For matched samples analysis, only data from subjects with both pre- and post-intervention RNA samples were included. A total of 29,663 probesets with unique gene annotation were selected out of 54,676 raw probesets for analysis. Analysis of Covariance (ANCOVA) was performed for both unmatched samples analysis and matched samples analysis. Batch and gender effects were adjusted for the unmatched sample analysis while only batch effect was adjusted in the matched sample analysis. Array data was normalized using (i) Microarray Suite 5 (MAS5) and subsequently log2 transformed, and (ii) Robust Multi-array Average (RMA) methods [5]. R software was used for normalization and differential gene expression analysis, and control of the false discovery rate (FDR) for multiple testing [6]. Gene expression is considered to show up-regulation in the post-intervention group if the adjusted fold change is more than 1.5 and downregulation if the adjusted fold change is less than 0.66. *P-values* < 0.05 (after FDR) were considered as statistically significant for differential gene expression (Table 1, Table 2, Table 3, Table 4, Table 5).

Hierarchical clustering was applied to both samples and signature gene set for (i) before FDR for pre-/post-HEP, (ii) pre-/post-MAP, (iii) after FDR and fold change cut-off (FC > 1.5 or FC < 0.66) for post-HEP/post-MAP, shown in the format of dendrogram in combination with a heatmap of gene expression levels.

### 2.5 Pathway analysis

The differentially expressed genes from MAS5 normalization were used for enrichment analysis of pathways and diseases using version 6.8 of DAVID [7]. The list of probesets from Table 1, Table 2 was entered into the web application (david.ncifcrf.gov), with “AFFYMETRIX_3PRIME_IVT_ID” selected as the gene identifier and Homo sapiens selected as the species. Diseases were identified by the GAD and OMIM databases, and biochemical pathway by KEGG. Gene enrichment in annotation terms was measured by modified Fisher's exact test (EASE score). Functional annotation chart was visualized using minimum of 2 count and maximum EASE score (*p*-value) of 0.5. EASE score (*p*-value) of < 0.05 was considered as statistically significant (Table 6, Table 7).
