# Supplementary material for: Dataset on gene expression in the elderly after Mindfulness Awareness Practice or Health Education Program
Source: Data Brief. 2018 Mar 26;18:902–12. doi: 10.1016/j.dib.2018.03.086 (PMC5996403; doi:10.1016/j.dib.2018.03.086)
Supplement: Supplementary file 1 — Supplementary material [file mmc1.pdf]

CONFLICT OF INTEREST DECLARATION

Manuscript title: Dataset on gene expression in the elderly after Mindfulness Awareness Practice or Health Education Program

Article type: Data article

Manuscript number: DIB-D-18-00119

Authors: Hwee-Woon Lim, Woei-Yuh Saw, Lei Feng, Yuan-Kun Lee, Ratha Mahendran, Irwin Kee-Mun Cheah, Iris Rawtaer, Alan Prem Kumar, Ee-Heok Kua, Rathi Mahendran, Ene-Choo Tan

We confirm that the manuscript has been read and approved by all named authors.

Declarations of interest: none

We certify that the work that is reported in the manuscript has not received any financial support from any pharmaceutical company or other commercial interest. We further confirm that all authors declare no conflict of interest.

Sign on behalf of all authors

[signature] 15 Mar 2018

Date

Ene-Choo Tan,
Corresponding author
KK Research Centre,
KK Women's and Children's Hospital,
100 Bukit Timah Road, Singapore 229899
E-mail address: tan.ene.choo@kkh.com.sg, tanec@bigfoot.com
